# Content Analysis of Apps for Growth Monitoring and Growth Hormone Treatment: Systematic Search in the Android App Store

**DOI:** 10.2196/16208

**Published:** 2020-02-18

**Authors:** Luis Fernandez-Luque, José I Labarta, Ella Palmer, Ekaterina Koledova

**Affiliations:** 1 Qatar Computing Research Institute Doha Qatar; 2 Salumedia Labs Sevilla Spain; 3 Department of Pediatrics Hospital Universitario Miguel Servet Zaragoza Spain; 4 inScience Communications Springer Healthcare Ltd London United Kingdom; 5 Merck KGaA Darmstadt Germany

**Keywords:** growth hormone, telemedicine, growth monitoring, mobile app, mobile health

## Abstract

**Background:**

The use of mobile apps for health is growing. This rapid growth in the number of health apps can make it hard to assess their quality and features. The increased demand for and availability of mobile health apps highlights the importance of regular publication of reviews to identify potential areas of unmet needs and concern. The focus of this review is mobile apps for monitoring growth for health care professionals, caregivers, and patients. Monitoring growth as a part of healthy physical development is important across different periods of childhood and adolescence.

**Objective:**

The goal of this content analysis is to map and understand the types of apps that currently exist that are related to growth monitoring and growth hormone treatment.

**Methods:**

A semiautomated search was undertaken using the app search engine 42Matters, complemented by a manual search for growth apps using the web search tool of Google Play (Android App Store). Apps were rated on their relevance to growth monitoring and categorized by independent raters.

**Results:**

In total, 76 apps were rated relevant to growth monitoring or growth hormone treatment. The level of agreement was measured for the semiautomated search and was very high (Κ=0.97). The target audience for 87% of the apps (66/76) was patients and relatives, followed by health care professionals (11%; 8/76) and both (3%; 2/76). Apps in the category “growth tracking tools for children and babies” were retrieved most often (46%; 35/76) followed by “general baby care apps” (32%; 24/76), “nonpharmacological solutions for growth” (12%; 9/76) and “growth hormone–related” (11%; 8/76). Overall, 19/76 apps (25%) tracked a precise location.

**Conclusions:**

This study mapped the type of apps currently available for growth monitoring or growth hormone treatment that can be used as a foundation for more detailed evaluations of app quality. The popularity of care apps for children and growth monitoring apps should provide a great channel for potential intervention in childhood health in the future.

## Introduction

The use of mobile apps for health is growing, and between 2008-2015, the growth in the number of apps used was quadratic [1]. In November 2017, according to IQVIA (formerly Quintiles and IMS Health, Inc), 318,000 apps were available worldwide with more than 200 health apps being added to the Apple Store and the Google Play store each day [1,2]. This rapid growth in the number of health apps can make it hard to assess their quality and features, although tools are being developed for this purpose [3], such as the Mobile App Rating Scale [4].

The increased demand for and availability of mobile health apps highlights the importance of regular publication of reviews to identify potential areas of unmet needs and concern. Consequently, methods for the systematic search for apps in app stores have been developed [5], and apps have been analyzed and characterized in disease areas where their use is widespread to identify their benefits and any shortcomings. For example, apps that focus on breast cancer have been shown to improve quality of life, and decrease stress [6]; however, it was found to be essential that medical personnel be involved in the creation of these apps to avoid the misuse of alternative therapies not supported by substantial evidence of efficacy [7]. Apps that focused on multiple sclerosis failed to meet patient needs and demands, and therefore, design collaboration between health professionals, researchers, and industry partners was suggested to increase patient adoption and engagement [8]. In a further analysis of multiple sclerosis physical activity apps, realistic goal setting and feedback were found to be critical for adoption [9]. In the field of diabetes, gaps between evidence-based recommendations and functionality of apps were revealed, potentially due to personalized education being underrepresented [10]. The conclusion of an evaluation of apps for endocrine-related disorders was that quality, content, data security, and privacy of apps were often low [11].

The privacy and data security of mobile health apps is an area of growing concern [12]. For example, there are some concerns that apps may be customized to retrieve extra personal information, such as GPS location, without the user’s knowledge [13]. This has led to different approaches emerging to help ensure that mobile health apps are safe for users. This includes national efforts, such as the UK National Health Service Health Apps Library only including apps that comply with UK data protection principles concerning information privacy [12]. Scalable systems have also been developed for analyzing and predicting Android app compliance with privacy requirements [14].

Further development in mobile health apps has led to the concept of self-monitoring, which is becoming more widespread with the advent of devices such as Fitbits and Apple Watches. Fitbits can measure fitness levels and sleep patterns, and the Apple Watch can monitor Hemoglobin A_1c_ in diabetes patients [15] and heart rate/electrocardiogram in patients with cardiovascular diseases [16] to reveal anomalies, such as risk of arrhythmias. Other examples of self-monitoring include personal recording of blood pressure for potential hypertension [17] and the use of real-world glucose monitoring devices connected to the web by diabetes patients [18].

Monitoring growth as a part of healthy physical development is part of preventive child health programs, as growth restriction and short stature are regarded as relatively early signs of poor health. Height measurements in pediatric populations, generally referred to as growth monitoring, can theoretically identify treatable conditions in apparently healthy short children. Early diagnosis of growth disorders is essential for prognosis, and it should be the primary objective of pediatric endocrinologists as it can benefit the patient and avoid or diminish the complications of an unrecognized disorder [19,20]. Growth monitoring is also very important in children with growth hormone deficiency, children who are small for their gestational age, or for syndromes requiring treatment with growth hormone, as it can help to identify the condition early enough to improve the prognosis, predict growth outcomes [21], and evaluate adherence and response to treatment [22]. Health care professionals can monitor children’s growth via adherence to growth hormone treatment through web-based platforms [23,24] and professional apps, which include official charts from organizations such as the World Health Organization [25]. Automated growth monitoring, where algorithms are integrated into electronic health records, is more efficient than standard growth monitoring, with a higher rate of referral to specialists and higher diagnostic yield of growth disorders. It has been shown that the prevalence of pathological short stature among referred children increased from 5.9% to 13.4% with this form of monitoring [26].

The focus of this review is mobile apps for monitoring growth for health care professionals, caregivers, and patients. Critical factors for the usability of growth apps are yet to be determined; therefore, this paper aims to provide a practical use and information overview for clinicians.

## Methods

### Extraction of Information About Health Apps Related to Growth

**Figure 1:**
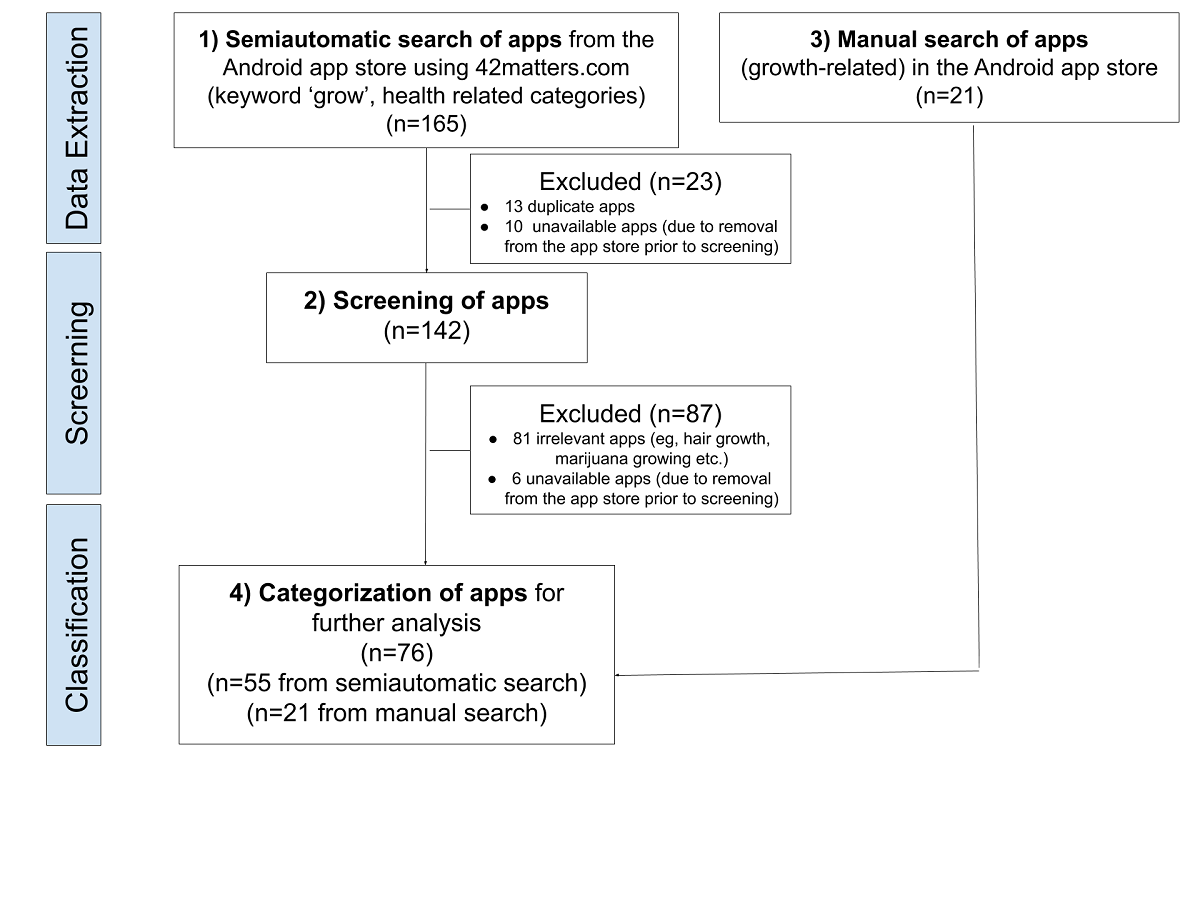
Study flow.

For the semiautomated search, as shown in Figure 1, searches were undertaken using the app search engine, 42Matters [27]. The use of 42Matters for obtaining information about health apps has previously been reported on in the literature [7,28,29]. Within 42Matters, we used the keyword “grow” to extract apps from the Android app store (Google Play) [30] in the Medical, Health, and Parenting categories. Data retrieved included the description, rating score, number of downloads, price, URL, and permission information.

The automated search using 42Matters was complemented with a manual search for growth apps using the web search tool of Google Play and using the terms “Growth hormone,” “Growth,” “Height,” and “Short stature”. The search was not limited by language. This manual search was undertaken to ensure all relevant apps were identified, since the availability of mobile apps can vary for technical reasons (eg, the temporal unavailability of apps, limitation to specific geographical regions).

The lists from the semiautomatic and manual searches were combined, and duplicate apps removed. Extended metadata (eg, permissions, downloads, screenshots) were extracted using a web-crawler and the 42Matters application programming interface. An updated version of the code used for retrieving the apps is available at GitHub under the creative commons license [31]. Standard and professional versions of apps were included.

### Screening

The descriptions of the automatically extracted apps were read by two independent raters (LFL and EP) who gave the apps a “relevant” score (1) if the app was specific to growth monitoring or growth hormone treatment, or a “not relevant” score (0) if it was not. The level of agreement between the two raters was measured by an interrater score calculated using Cohen’s kappa [32]. Cohen’s kappa was calculated using the R Statistical Framework package IRR (R Foundation, Vienna, Austria). Ratings were: <0.20, poor; 0.21-0.40, fair; 0.41-0.60, moderate; 0.61-0.80, good; 0.81-1.00, very good.

### Classification

The apps were classified further in a meeting between clinical and electronic health experts. Several growth-related applications were discussed, and a subset of categories was agreed upon and tested by three researchers (LFL, EK, and JC) using a random sample of growth-related apps. In a final consensus meeting, a complete set of categories were selected, and a categorization form was developed and tested for usability and relevance with a further random sample of growth-related apps. The final categorization form was hosted by Google Forms [33].

Three independent raters (LFL, EK and JC) then categorized the apps by reading the description of the app and filling in the categorization form as follows: (1) Target Audience (one choice from Patients/caregivers, Health care professionals, both, uncertain); (2) Type of app (one choice from growth tracking for children and babies, general baby care apps, growth hormone–related, nonpharmacological solutions for growth, unclear); (3) presence of medical/scientific references; and (4) additional aspects included in the app (multi-choice) (see Multimedia Appendix 1). If any of the classifications was not clear, a consensus was reached at a meeting with LFL and EP in January 2019. All analyses were reported descriptively.

## Results

### Overview

Information about health apps related to growth was extracted in a number of steps (Figure 1). Initially, the keyword “grow” was used to retrieve 142 apps from the app search engine of 42matters [27].

### Screening

In total, 76 apps were rated “relevant” to growth monitoring or growth hormone treatment, with 55 from the semiautomated search and 21 from the manual search. The level of agreement between the two raters was measured for the semiautomated search and was very high (Κ=0.97), with just three discrepant results, which were resolved at a consensus meeting.

**Figure 2:**
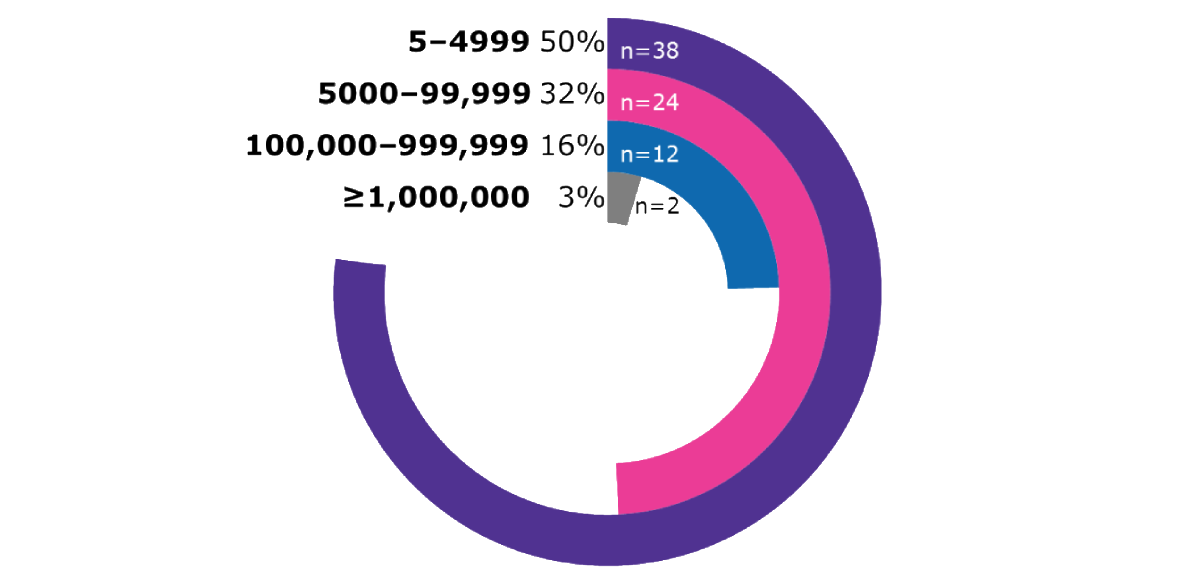
Percentage of apps in each download range.

Cumulatively, there were at least 3.75 million downloads of the apps (Figure 2). The majority of apps (50%; 38/76) had between 5 and 4999 downloads, whereas just two apps (3%) had more than 1,000,000 downloads. It should be noted that while download numbers are a useful indicator of the popularity of apps, they should be used with caution. Some apps are not available in all countries, some may only be usable within a given hospital, and the number of downloads is greatly affected by the amount of time the app has been available.

### Classification and Analysis

**Figure 3:**
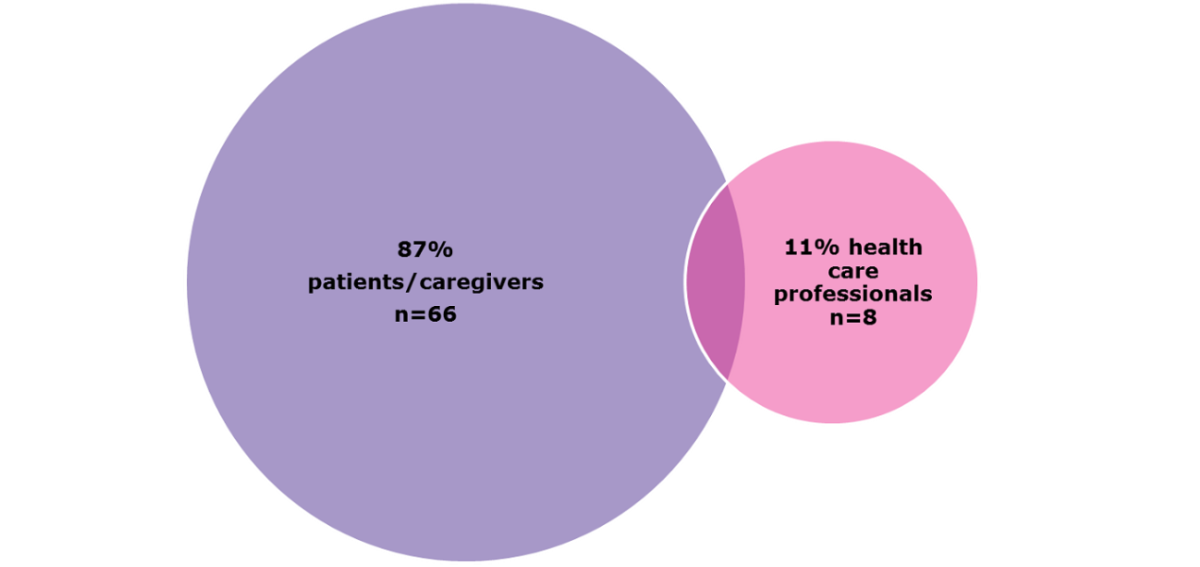
Percentage of growth-related apps by target audience.

The target audience for 87% of the apps (66/76) was patients and relatives. Health care professionals were the target audience for 11% of the apps (8/76), and two apps were considered to fall into both patient/relative and health care professional categories (Figure 3).

**Figure 4:**
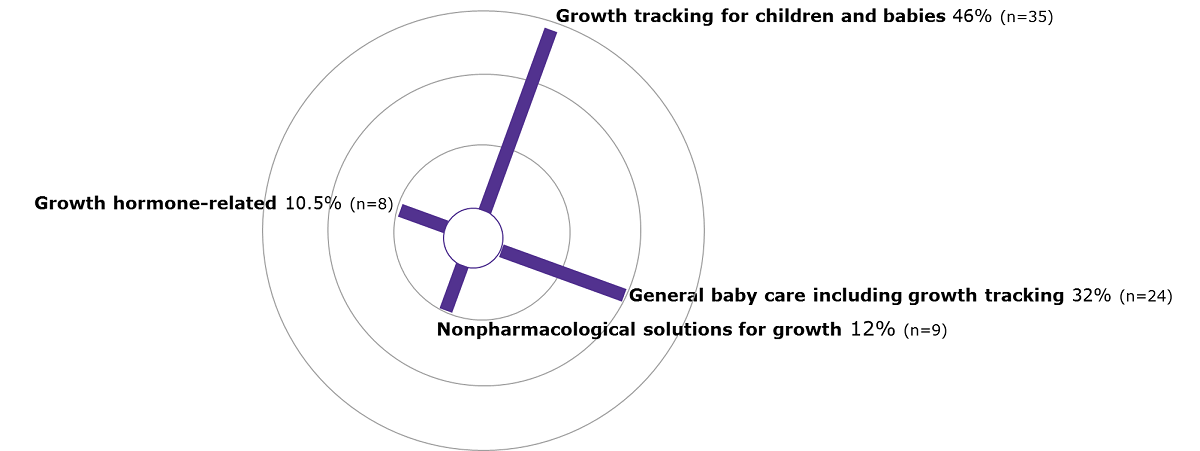
Percentage of apps in each growth tool category. Percentages have been rounded up/down, therefore do not total 100%.

Apps in the category of growth tracking tools for children and babies were retrieved most often (46%; 35/76), followed by general baby care apps (32%; 24/76). These were apps which combined growth tracking with gamification and personalization features, such as inclusion of family photos and records of daily routines (eg, feeding). Nonpharmacological solutions for growth comprised 12% of the apps (9 /76), and 11% of the apps were growth hormone–related (8/76) (Figure 4 and Multimedia Appendix 2).

Overall, 31% (11/35) of apps in the growth tracking tools for children and babies category included references to growth charts, as did 46% (11/24) of apps in the general baby care category, and 13% (1/8) of apps in the growth hormone category. However, no apps in the nonpharmacological solutions for growth category included references to growth charts (see Multimedia Appendix 2).

The eight growth hormone–related apps were all targeted at patients/caregivers. They all included education about growth hormone deficiency and related diseases, and four apps also included education about growth tracking (eg, referral to correct growth references) or supporting and tracking adherence (Multimedia Appendix 3). Claims within the description of the growth hormone apps were aimed at improving quality of life; “these records can then be shown to a doctor who can in turn use them to improve the patients’ quality of life” and “receive reminders on when to inject, track adherence history, monitor growth and better connect with the care team.”

Apps describing nonpharmacological solutions for growth included “natural” growth treatments for adults, and included claims in the description such as “let this guide provide peace of mind that you can actually grow 1 to 4 inches through these techniques” and ”grow your height by using our app” (Multimedia Appendix 4).

**Figure 5:**
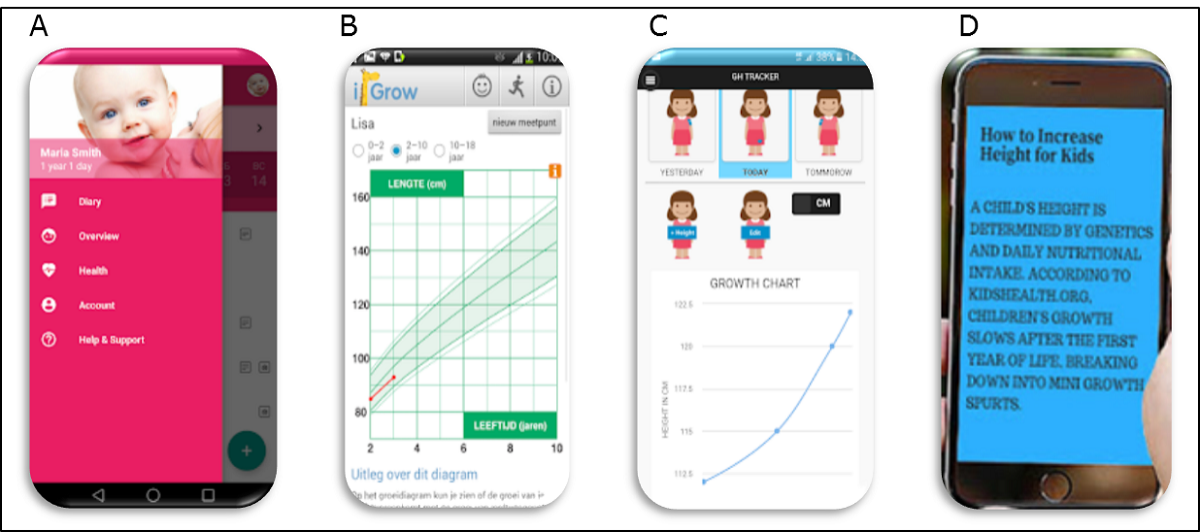
Examples of app interface for each category. A. General baby care apps; B. Growth Tracking for children and babies; C. Growth hormone apps; and D. Nonpharmacological solutions for growth.

The visual appearance of the apps varied considerably (Figure 5). The general baby care apps included visually appealing images such as a baby’s face, apps including growth tracking tools and growth hormone apps included images such as graphs, and the nonpharmacological apps were often mostly text with simple or no images.

Permission data was extracted. A total of 19/76 apps (25%) tracked a precise location: 5/35 (14%) of the “growth tracking for children and babies” apps, 9/24 (38%) of the “general baby care” apps, which include growth tracking tools, 1/8 (13%) of the growth hormone apps, and 4/9 of the nonpharmacological treatment for growth apps (44%).

## Discussion

This study is the first to map and understand the type of apps currently available that are related to growth monitoring. The majority of growth apps were general baby care apps, which include growth tracking tools. Growth tracking apps for children and babies were also popular, whereas there were fewer nonpharmacological and growth hormone apps. Patients and caregivers were the target audience for the vast majority of the apps, and the remainder were targeted at health care professionals or both. The quality of the apps was very heterogeneous, and in general, baby care apps were focused on monitoring and growth hormone apps were focused on education. Almost a quarter of the growth apps tracked precise location, which raises potential concerns in terms of privacy.

There is great interest in apps for monitoring growth and growth hormone treatment, as evidenced by the high download numbers in this evaluation. However, as observed in the first filtering of this analysis, two-thirds of apps were not relevant, indicating that navigating the range of available apps is not a trivial task. A high proportion of apps were aimed at growth in children and babies, aligning with the stage when growth is mostly dependent on nutrition (generally up to 4 years old) [34]. There were also nonpharmacological apps generally aimed at adults, which tended to prey on insecurities about short stature and offer advice not always backed up by scientific evidence.

Privacy is of particular concern, as noted in this evaluation. Some of the growth apps, such as 4/9 apps with nonpharmacological treatments for growth, requested location permissions without any apparent reason. One potential explanation is that location-based advertisements placed in mobile apps can be personalized and, therefore, apps made more profitable.

There is a need to educate users and health care professionals on digital health literacy and how to evaluate the trustworthiness of apps using tools that evaluate quality [3,4] and privacy settings [12,14]. Although there are frameworks for digital health literacy [35], our findings with regard to accessing private information, such as location, highlight the need to reinforce privacy and safety training to help clinicians and patients make safe decisions when choosing health apps. There is very little data on the assessment of apps in peer-reviewed publications, and there is a need for a list of useful and validated apps as well as an outlet for feedback on apps that include unrealistic expectations or inaccurate information.

More research is needed on how to facilitate the prescription of health apps [36], as it can be a time-consuming effort for health care professionals. Further, previous research [12] has shown that severe privacy risks have been found in a white list of apps recommended by health authorities. We should be aware that any assessment of the privacy and data security aspects of a mobile health app, like any other quality aspect, might vary when a new updated version is made public. A potential approach to overcome the dynamic nature of mobile health is to invest more in increasing digital literacy skills.

As shown in this analysis and other studies [7,10], there are currently few apps that include education and links between users and health care professionals. To allow users to detect abnormal growth and seek appropriate medical advice and care early on, an ideal app should combine educational and reference targets and be based on input from users such as parents and older children, as well as health care professionals. With growth disorders, similar to diabetes, optimizing health care delivery through apps is essential, as patients spend much time administering treatment by themselves without health care professional input. Apps for patients/caregivers should, therefore, support information exchange with health care professionals and patient support groups. All users of growth apps should receive educational information that includes the importance of attaining the objectives of growth hormone treatment during infancy, such as normalization of height as early as possible, maintaining normal height velocity, and attainment of normal adult height consistent with parental height.

Treatment adherence remains the most important factor influencing successful outcomes, as in most chronic therapies, including growth disorders [37,38]. Low adherence to growth hormone treatment is a significant factor determining reduced growth gain along with increased health costs. Several strategies have been proposed to improve patient adherence and education of parents and patients is essential [39]. Baby care apps often fail to provide adequate information on normal growth and development later in childhood and adolescence, or how to detect abnormal growth at an early stage and obtain medical advice, and, where necessary, care and treatment. There is also an unmet educational need for the prediction of growth outcomes to avoid unrealistic expectations. Apps developed by scientific and professional bodies, such as the “grow on the go” app, should be used by developers as a gold standard model, with validation by experts in auxology for accuracy and adequacy of growth charts.

Complementary to the methods for the systematic search of apps in app stores [5] that have been published, this review also explores a semiautomated approach to extract mobile app data, which can be used to facilitate part of the process of the analysis of health apps. Additionally, we observed a disparity in the availability of apps between the manual and semiautomated searches, which can be due to reasons such as versions of the mobile apps not being available in a given country and temporary removal of apps in the store. Commercial search engines for apps do not disclose their algorithms, and for that reason, we advocate complementing manual searches with automated approaches to minimize the risk of missing relevant apps. In our analysis of health apps, we only analyzed the description and screenshots of the health apps, not the ratings or the comments, as this would have been very time-consuming in a manual content analysis. However, new app-mining techniques are an emerging area which could help to automatize apps for security [40], user reviews [41], features [42], gamification [43], and usability [44], among other aspects.

This initial analysis of growth-related apps was undertaken in the Android app store Google Play [30] as it currently has the highest number of apps available [45]. An analysis of iOS, Oppo, and Huawei apps was not undertaken due to difficulties extracting permission data, such as location tracking. Other limitations of this evaluation are that the number of downloads might not be correlated to usage and that some apps might have launched in only a few countries and, therefore, not visible in this screen. Apps were not downloaded for this review, which means some features and screenshots not visible in the description may have been missed. Additionally, usability [44] was not addressed, as it is extremely resource-intensive, especially in the context of growth since some apps are directed towards multiple groups (ie, children, patients, and clinicians). We also did not study elements related to user experiences, such as gamification and personalization. This is something of great importance that has been widely researched in other health areas [7,43].

Additionally, our report on privacy issues is very limited. For example, we did not analyze the privacy policy of the applications. However, clinicians should be aware of recent studies highlighting serious concerns on many health apps [46,47]. Further, clinicians and patients should be aware that available health apps might comply with the regulations in one country and not others.

This evaluation has shown that the vast majority of apps focused on the growth of babies and young children, with far fewer nonpharmacological apps and growth hormone apps available. Patients and caregivers were the target audience for most of the apps. The popularity of care apps for children and growth tracking apps should provide a great channel for potential intervention in childhood health in the future. Families can be empowered through mobile apps as they can monitor height against references [25] and, if taking growth hormone, can monitor their height and adherence in close cooperation with their health care team. Including education about the importance of growth potential and outcomes in childhood, apps could help combat misleading advice in growth apps aimed at adults. Further evaluations should include iOS apps, and a detailed study of the quality of existing health apps related to growth should be undertaken.

## Appendix

Multimedia Appendix 1 Categorization form.

Multimedia Appendix 2 Table A. Apps targeted at a healthcare professional audience. Table B. Apps targeted at a patient/caregiver audience.

Multimedia Appendix 3 Growth tracking features of growth hormone apps.

Multimedia Appendix 4 Apps with non-pharmacological solutions for growth.
