# Supplementary figures and images for: Content Analysis of Apps for Growth Monitoring and Growth Hormone Treatment: Systematic Search in the Android App Store
Source: JMIR Mhealth Uhealth. 2020 Feb 18;8(2):e16208. doi: 10.2196/16208 (PMC7055837; doi:10.2196/16208)

**Multimedia Appendix 1. Categorization form**

#
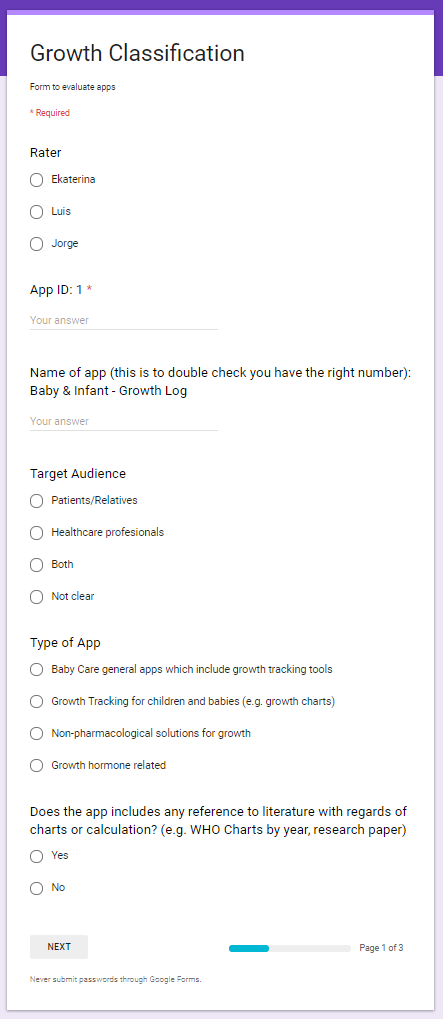

Supplement: Multimedia Appendix 1 [file mhealth_v8i2e16208_app1.docx]
