# Supplementary material for: Content Analysis of Apps for Growth Monitoring and Growth Hormone Treatment: Systematic Search in the Android App Store
Source: JMIR Mhealth Uhealth. 2020 Feb 18;8(2):e16208. doi: 10.2196/16208 (PMC7055837; doi:10.2196/16208)
Supplement: Multimedia Appendix 2 [file mhealth_v8i2e16208_app2.docx]

# Multimedia Appendix 2

**Table A. Apps targeted at a healthcare professional audience**

| **No. of downloads (range)** | **App name** | **Example screenshot** | **Type of app** | **Include references to growth charts?** | **Specific app features** |
| --- | --- | --- | --- | --- | --- |
| 100,000–499,999 | Ped(z) - Pediatric Calculator | 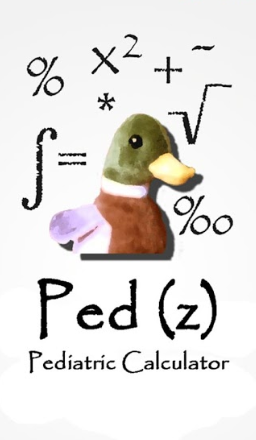 | Growth tracking for child/baby | Yes | Growth prediction tools (e.g. target height) |
| 50,000–99,999 | Growth Chart CDC WHO Percentil | 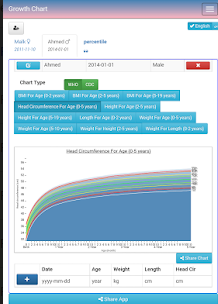 | Growth tracking for child/baby | No | – |
| 10,000–49,999 | Growth Charts UK-WHO | 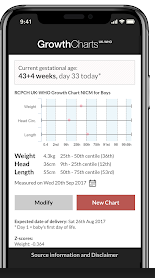 | Growth tracking for child/baby | Yes | – |
|  | IAP Growth Chart Application | 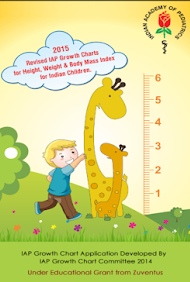 | Growth tracking for child/baby | No | – |
|  | Percentil Calculator | 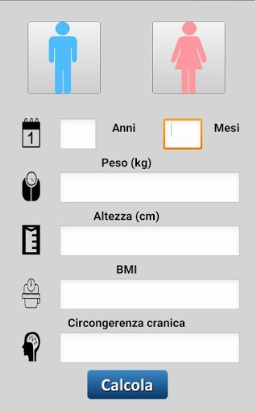 | Growth tracking for child/baby | Yes | – |
| 5,000–9,999 | Baby Growth Standards | 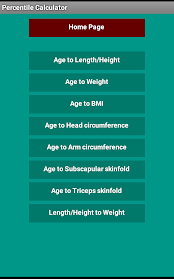 | Growth tracking for child/baby | No | – |
| 1,000–4,999 | MQ Growth - OMS escore z PT-BR | 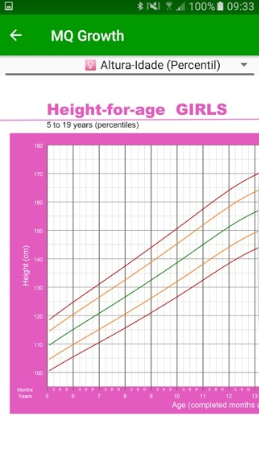 | Growth tracking for child/baby | No | – |
|  | WHO growth standards | 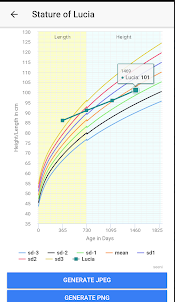 | Growth tracking for child/baby | No | Endocrine examination (puberty, bone age, parental height) |
| 100–999 | Growth Standards | 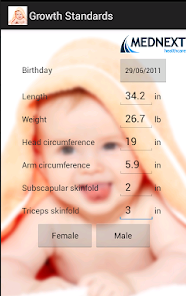 | Growth tracking for child/baby | Yes | – |
| 10–99 | WHOZ Scores based Child Growth Tracker | 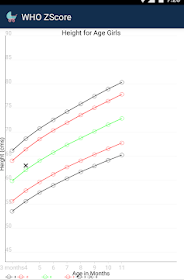 | Growth tracking for child/baby | No | – |

**Table B.** **Apps targeted at a patient/caregiver audience**

| **No. of downloads (range)** | **App name** | **Example screenshot** | **Type of app** | **Include references to growth charts?** | **Specific app features** |
| --- | --- | --- | --- | --- | --- |
| ≥1,000,000 | Baby Care - track baby growth! | 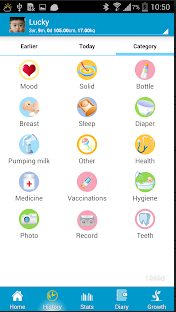 | General baby care with growth tracking tool | No | – |
|  | Peekaboo Moments – Babybook, memories & moments | 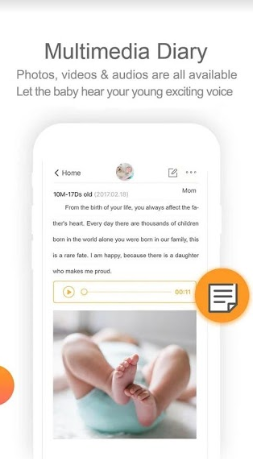 | General baby care with growth tracking tool | Yes | Growth prediction tools (e.g. target height) |
| 500,000–999,999 | Baby + – your baby tracker | 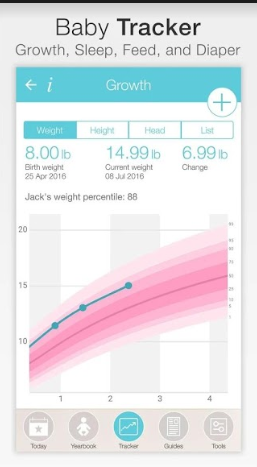 | General baby care with growth tracking tool | Yes | Growth tracking education |
|  | Baby Daybook - Breastfeeding & Care Tracker | 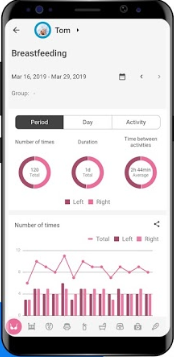 | General baby care with growth tracking tool | Yes | Growth tracking education |
|  | Baby Tracker - Newborn Feeding, Diaper, Sleep Log | 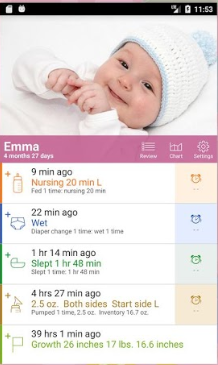 | General baby care with growth tracking tool | Yes | Growth tracking education |
|  | WebMD Baby | 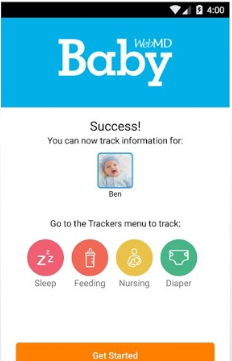 | General baby care with growth tracking tool | No | – |
| 100,000–499,999 | Child Growth & Percentiles | 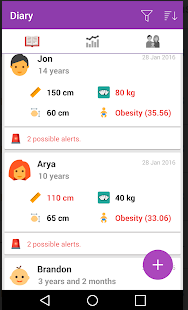 | Growth tracking for child/baby | No | Growth prediction tools  Growth tracking education |
|  | uGrow baby development tracker | 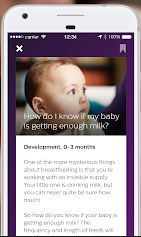 | General baby care with growth tracking tool | No | – |
|  | Baby Care Tracker - Breastfeeding | 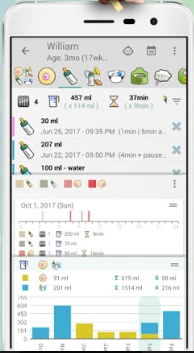 | General baby care with growth tracking tool | Yes | Growth tracking education |
|  | Babygogo Parenting - Baby & Mothercare App | 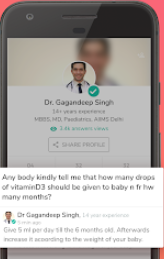 | General baby care with growth tracking tool | No | – |
|  | Glow Baby Breastfeeding Tracker, Nursing Timer App | 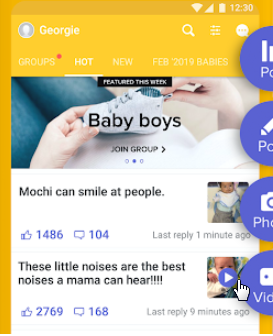 | General baby care with growth tracking tool | No | – |
|  | Indian Pregnancy & Parenting Tips,The Babycare App | 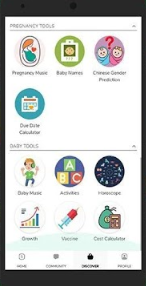 | General baby care with growth tracking tool | No | – |
|  | Child Growth Tracker | 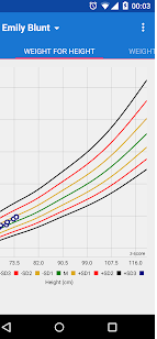 | Growth tracking for child/baby | Yes | – |
| 50,000–99,999 | Child Growth Standards | 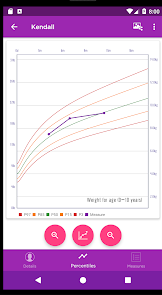 | Growth tracking for child/baby | No | Growth tracking education |
|  | Growth Chart Trial | 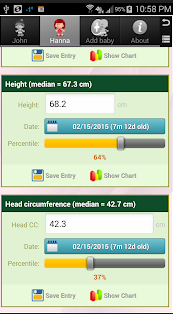 | Growth tracking for child/baby | No | – |
|  | Feed Baby Pro - Baby Tracker | 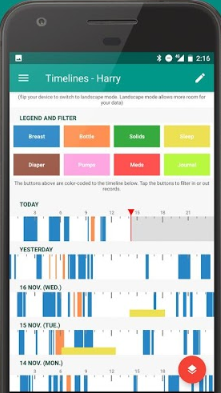 | General baby care with growth tracking tool | Yes | Growth tracking education |
| 10,000–49,999 | Baby Growth and Care Breastfeeding | 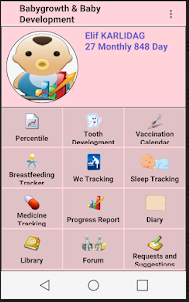 | General baby care with growth tracking tool | No | – |
|  | Baby Growth Chart | 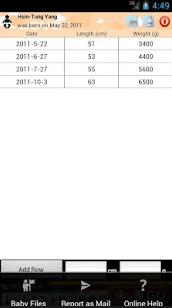 | Growth tracking for child/baby | No | – |
|  | Baby Journal: Child Growth & Milestone Book | 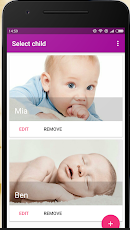 | General baby care with growth tracking tool | No | – |
|  | Child Growth Tracker | 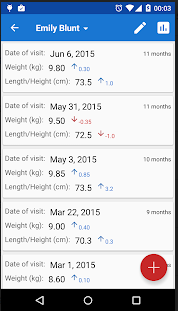 | Growth tracking for child/baby | No | Growth tracking education |
|  | IAP Growth Chart Application | 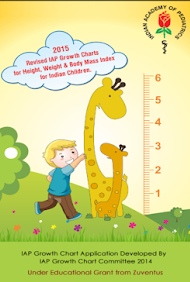 | Growth tracking for child/baby | No | – |
|  | iGrow | 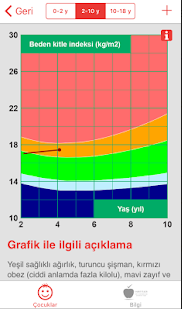 | General baby care with growth tracking tool | Yes | – |
|  | iGrow, de groei app van TNO | 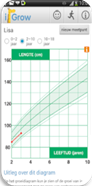 | Growth tracking for child/baby | Yes | Growth tracking education |
|  | Baby Percentile | 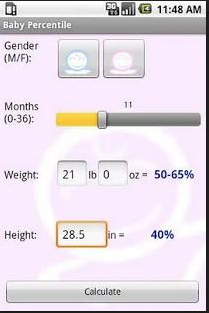 | General baby care with growth tracking tool | No | – |
|  | Babylog (Parenting, Track & Analysis) | 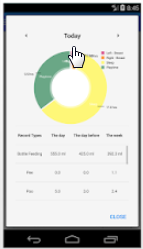 | General baby care with growth tracking tool | Yes | – |
|  | Percentil Calculator | 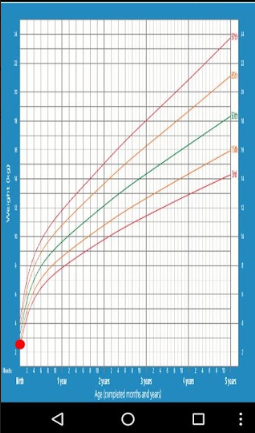 | Growth tracking for child/baby | Yes | – |
| 5,000–9,999 | Baby Development - Growth Log | 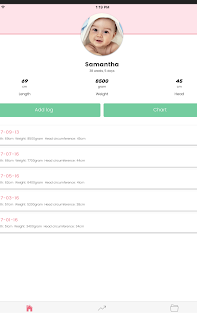 | Growth tracking for child/baby | Yes | Growth tracking education |
|  | Baby Growth chart WHO | 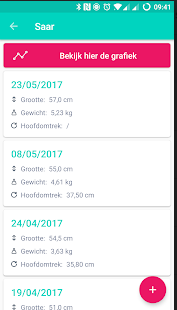 | Growth tracking for child/baby | No | Growth tracking education |
|  | Grow Taller Fast | 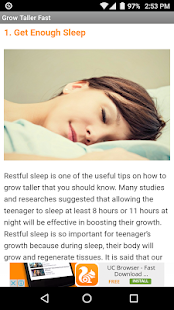 | Non-pharmacological solutions for growth | No | – |
|  | Growth Chart | 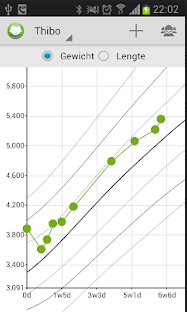 | Growth tracking for child/baby | No | – |
|  | Height Growth | 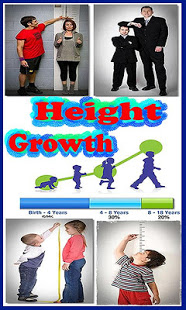 | Non-pharmacological solutions for growth | No | Growth prediction tools |
|  | InLab: Growth Diary | 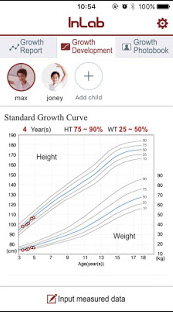 | Growth tracking for child/baby | No | Growth tracking education |
|  | Baby Care Plus | 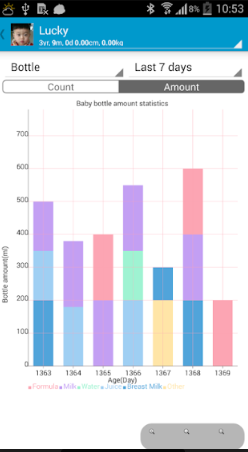 | General baby care with growth tracking tool | No | – |
|  | baby Growth Chart WHO | 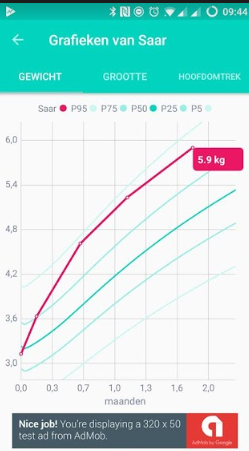 | General baby care with growth tracking tool | Yes | Growth prediction tools |
| 1,000–4,999 | Baby Growth Calculator | 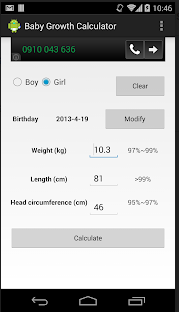 | Growth tracking for child/baby | Yes | Growth tracking education |
|  | Baby Tracker. Breastfeeding Log & Nursing - MeGrow | 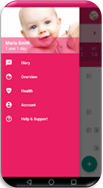 | General baby care with growth tracking tool | Yes | – |
|  | Child Growth Diary | 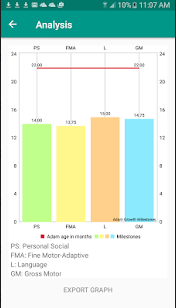 | General baby care with growth tracking tool | Yes | Growth tracking education |
|  | Child Growth Tracker | 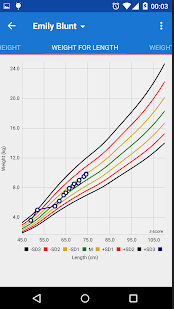 | Growth tracking for child/baby | Yes | Growth tracking education |
|  | Child Growth Tracker(BMI) | 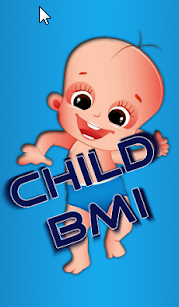 | Tools for specialists | No | – |
|  | Grow Taller Guide | 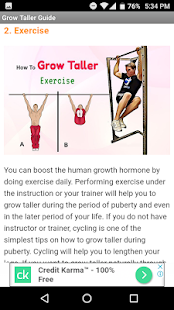 | Non-pharmacological solutions for growth | No | – |
|  | growin | 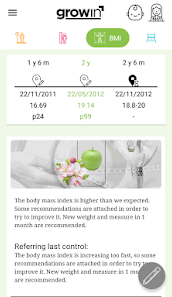 | Growth tracking for child/baby | No | GHD education  Growth prediction tools  Growth tracking education |
|  | Growth | 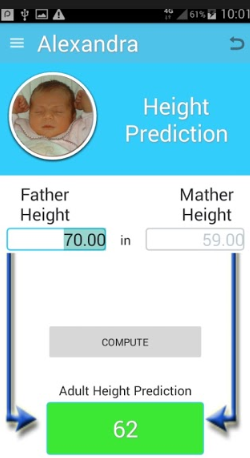 | Growth tracking for child/baby | Yes | Growth prediction tools  Growth tracking education |
|  | Growth Chart Pro | 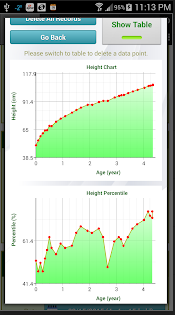 | Growth tracking for child/baby | No | Growth prediction tools |
|  | Growth Charts | 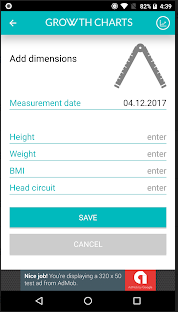 | Growth tracking for child/baby | No | – |
|  | growth Record ( Baby Graph ) | 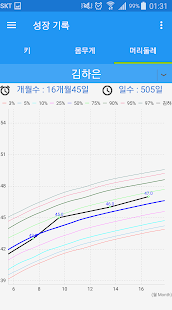 | Growth tracking for child/baby | No | – |
|  | How To Grow Taller | 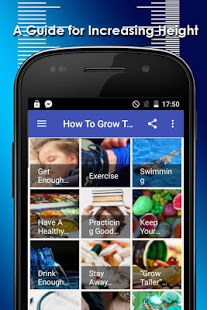 | Non-pharmacological solutions for growth | No | – |
|  | MamaTracker - baby growth log | 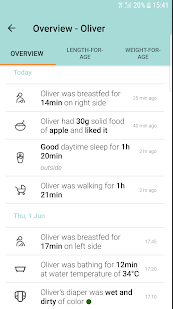 | General baby care with growth tracking tool | No | – |
| 500–999 | Baby Growth Tracker | 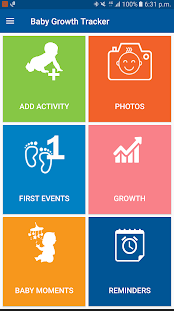 | General baby care with growth tracking tool | No | – |
|  | Growth Hormone Tracker | 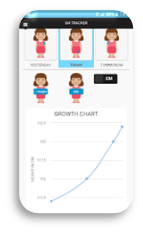 | Growth hormone related | No | Adherence support and tracking  GHD education  Growth tracking education |
| 100–499 | Baby Growth Calculator | 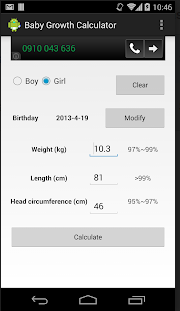 | Growth tracking for child/baby | No | – |
|  | BabyGrowing | 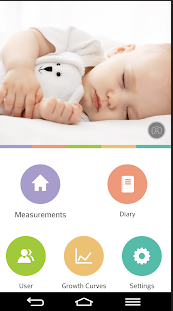 | Growth tracking for child/baby | No | – |
|  | CHILD GROWTH CALCULATOR | 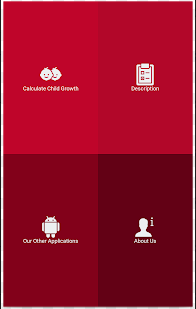 | Growth tracking for child/baby | No | Growth tracking education |
|  | eGrowth Monitoring | 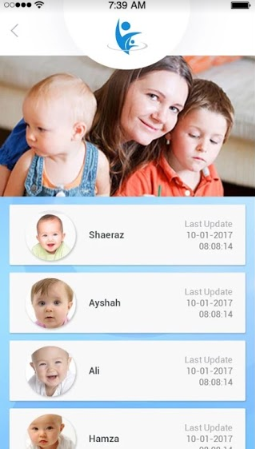 | Growth tracking for child/baby | No | Growth tracking education |
|  | Grow Taller Naturally | 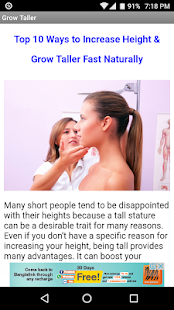 | Non-pharmacological solutions for growth | No | Detailed endocrine examination |
|  | Millennials' Growth | 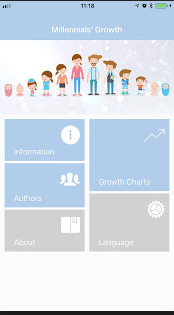 | Growth tracking for child/baby | Yes | Growth tracking education |
|  | Signs&Symptoms Growth Hormone | 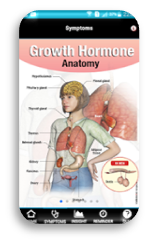 | Growth hormone related | No | GHD education |
|  | RASTIEM | 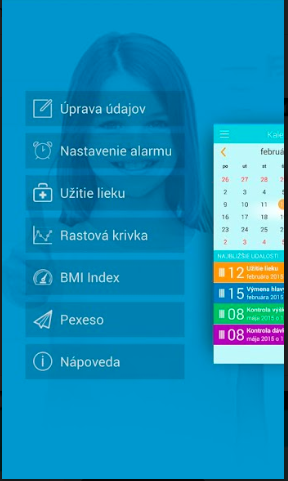 | Growth hormone related | No | Adherence support and tracking |
| 50–99 | Grow on the Go | 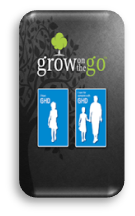 | Growth hormone related | Yes | GHD education |
| 10–49 | Child Growth Tracker Pro | 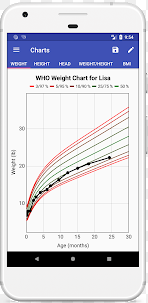 | Growth tracking for child/baby | No | – |
|  | Height Growing Tips | 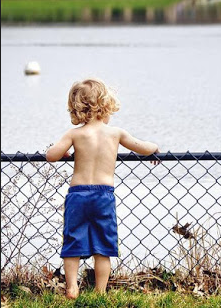 | Non-pharmacological solutions for growth | No | – |
|  | HOW TO GROW TALLER | 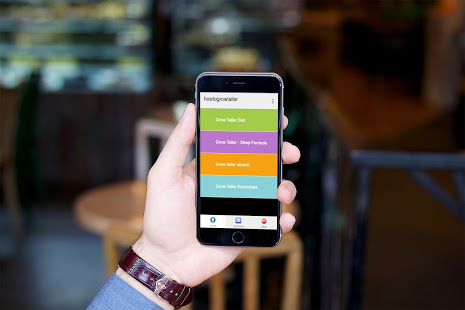 | Non-pharmacological solutions for growth | No | – |
|  | HOW TO GROW TALLER FAST | 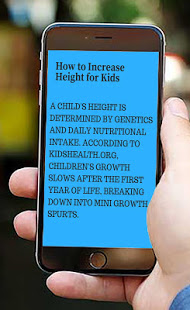 | Non-pharmacological solutions for growth | No | – |
|  | How To Grow Taller Naturally | 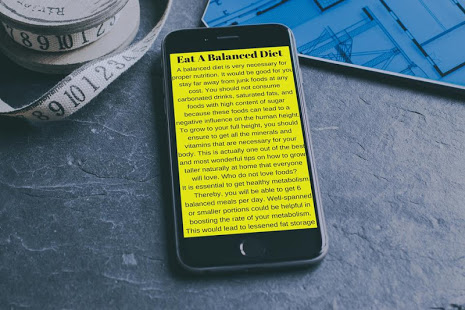 | Non-pharmacological solutions for growth | No | – |
|  | GroAssist Pfizer Inc. Medical | 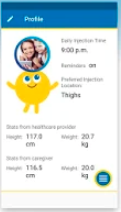 | Growth hormone related | No | GHD education  Adherence support and tracking |
|  | Vyrostu MixedApps.cz | 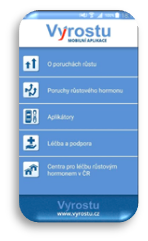 | Growth hormone related | No | GHD education |
| 5–9 | Baby Care - Track growth and Information | 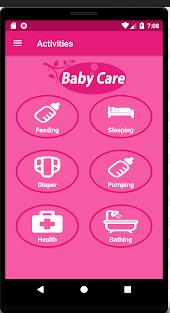 | General baby care with growth tracking tool | No | – |
|  | growlink app | 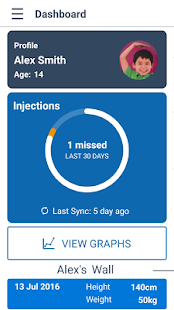 | Growth hormone related | No | Adherence support and tracking  GHD education  Growth tracking education |
|  | Saizoom | 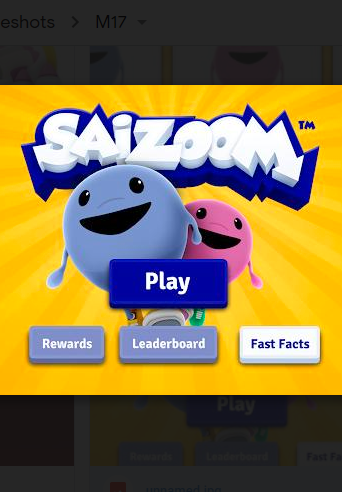 | Growth hormone related | No | GHD education |
