# Supplementary material for: Content Analysis of Apps for Growth Monitoring and Growth Hormone Treatment: Systematic Search in the Android App Store
Source: JMIR Mhealth Uhealth. 2020 Feb 18;8(2):e16208. doi: 10.2196/16208 (PMC7055837; doi:10.2196/16208)
Supplement: Multimedia Appendix 3 [file mhealth_v8i2e16208_app3.docx]

**Multimedia Appendix 3. Growth tracking features of growth hormone apps**

| **App name** | **Example screenshot** | **Features** | | |
| --- | --- | --- | --- | --- |
|  |  | **Education** | | **Support** |
|  |  | Growth hormone deficiency and related diseases | Growth tracking (e.g. data interpretation, referral to correct growth chart reference) | Tracking adherence to growth hormone treatment |
| Grow on the Go | 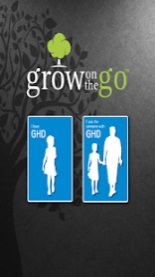 | X | – | – |
| Growth Hormone Tracker | 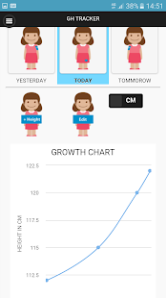 | X | X | X |
| Signs&Symptoms Growth Hormone | 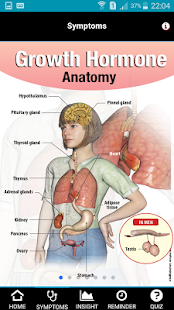 | X | – | – |
| growlink app | 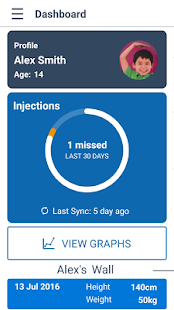 | X | X | X |
| Saizoom | 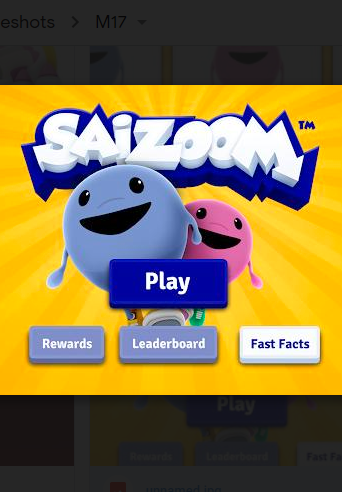 | X | – | – |
| RASTIEM | 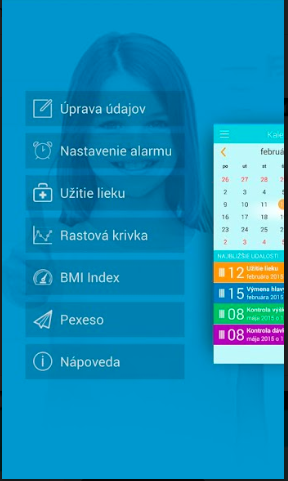 | X | X | X |
| GroAssist Pfizer Inc. Medical | 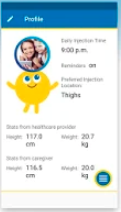 | X | – | X |
| Vyrostu MixedApps.cz | 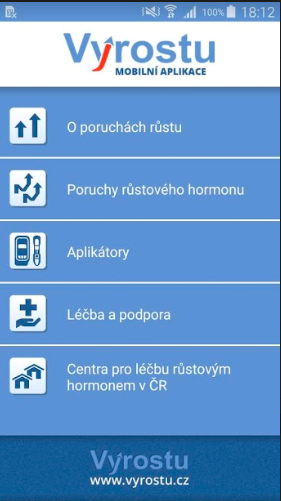 | X | – | – |
