# Supplementary material for: Content Analysis of Apps for Growth Monitoring and Growth Hormone Treatment: Systematic Search in the Android App Store
Source: JMIR Mhealth Uhealth. 2020 Feb 18;8(2):e16208. doi: 10.2196/16208 (PMC7055837; doi:10.2196/16208)
Supplement: Multimedia Appendix 4 [file mhealth_v8i2e16208_app4.docx]

**Multimedia Appendix 4. Apps with non-pharmacological solutions for growth.**

| **No. of downloads (range)** | **App name** | **Example screenshot** | **rating** | **Tips included in app description** |
| --- | --- | --- | --- | --- |
| 5,000–9.999 | Grow Taller Fast | 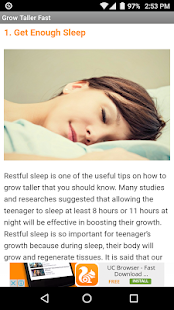 | 4.5 | - Get enough sleep - Have a healthy and well balanced diet - Practice good posture - Keep your immune system strong and healthy - **Drink 2 liters of water per day** |
|  | Height Growth | 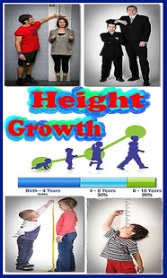 | 3.7 | Simple tips to increase height in a month:   - Daily stretching exercises - Swimming - Eat essential nutrients: zinc, manganese, vit. C, protein, potassium, calcium, phosphorus. - **Get vit. D from sunlight and milk** |
| 1,000–4,999 | Grow Taller Guide | 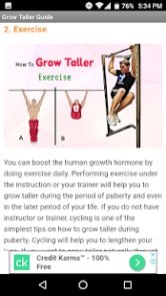 | 4.3 | Grow 1 to 4 inches through these techniques:   - Get enough sleep - **Exercise, particularly cycling** |
|  | How To Grow Taller | 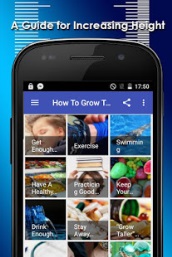 | 3.3 | - Get enough sleep - **Swimming** - Healthy and well balanced diet - Practice good posture |
| 100–999 | Grow Taller Naturally | 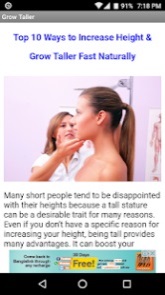 | 4.5 | - Get adequate sleep - Eat healthily - Get enough vitamin D and calcium - **Swim 2 hours per day** - **Hanging exercises** |
| 10–99 | HOW TO GROW TALLER FAST | 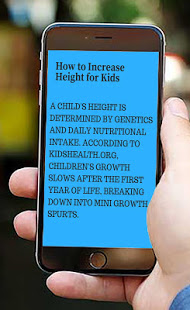 | NA | - Healthy diet for adults: fresh fruit and vegetables, lean proteins, complex carbohydrates, good fats - Healthy diet for children: Fish and lean meat |
|  | HOW TO GROW TALLER | 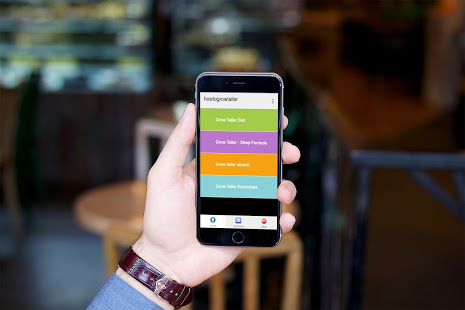 |  | - **Bar hanging** - **Dry land swim** - **Pelvic shift** - **Cobra stretch** - **Super cobra stretch** |
|  | How To Grow Taller Naturally | 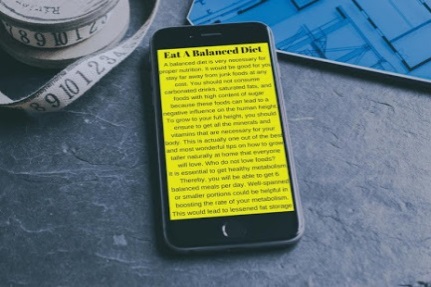 |  | Includes:   - Fruits for height growth - Foods for height growth |
|  | Height Growing Tips | 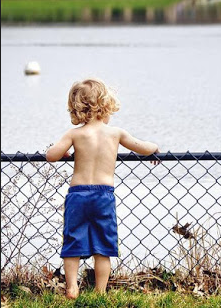 |  | - Eat a balanced diet - **Sprint fast** - Stretching exercises - High intensity exercises |
